# Ontario Veterinary Medical College Society

**Published:** 1896-01

**Authors:** H. R. Ryder

**Affiliations:** Secretary


### ONTARIO VETERINARY MEDICAL COLLEGE SOCIETY.

THIS Society, in connection with the Ontario Veterinary College, has held several meetings during this session, and a number of interesting and valuable papers have been presented. The officers are: Andrew Smith, F.R.C.V.S., President; H. R. Ryder, Secretary; T. V. Simpson, Assistant Secretary; G. R. C. Marriam, Treasurer; R. F. Hoadley, Librarian.

H. R. RYDER,
Secretary.

